# Excluding Lynch syndrome in a female patient with metachronous DNA mismatch repair deficient colon- and ovarian cancer

**DOI:** 10.1007/s10689-017-0055-1

**Published:** 2017-11-09

**Authors:** Stijn Crobach, Anne M. L. Jansen, Marjolein J. L. Ligtenberg, Marije Koopmans, Maartje Nielsen, Frederik J. Hes, Juul T. Wijnen, Winand N. M. Dinjens, Tom van Wezel, Hans Morreau

**Affiliations:** 1 Department of Pathology, Leiden University Medical Center, Leiden, The Netherlands; 2 Department of Human Genetics and Department of Pathology, Radboud University Medical Center, Nijmegen, The Netherlands; 3 Department of Clinical Genetics, Leiden University Medical Center, Leiden, The Netherlands; 4 Department of Human Genetics, Leiden University Medical Center, Leiden, The Netherlands; 5 Department of Pathology, Erasmus MC Cancer Institute, Rotterdam, The Netherlands; 6 Department of Pathology, Leiden University Medical Centre, Zone L1-Q, P.O. Box 9600, 2300 RC Leiden, The Netherlands

**Keywords:** Lynch syndrome, Ovarian cancer, Colon cancer

## Abstract

Patients synchronously or metachronously presenting with ovarian and colon cancer can pose diagnostic challenges. A primary colon carcinoma can metastasize to one or both ovaries, two independent primary tumors can arise or an ovarian carcinoma can metastasize to the colon. Clinical and immunohistochemical characterization can aid the diagnosis. Recently, we reported that in difficult cases finding pathogenic *APC* variants supports a colonic origin.

In this case report we describe the clinical history of a female patient suspected for Lynch syndrome. She was diagnosed with a bilateral ovarian cancer at age 44, followed by the detection of a colon carcinoma 12.5 months later. Lesions of both sites showed a DNA mismatch repair deficiency with immunohistochemical loss of MLH1 and PMS2 expression without *MLH1* promoter hypermethylation. In absence of germline MMR gene variants identical somatic *MLH1* and *CTNNB1* gene variants were found, indicating a clonal relation. MMR germline mosaicism was made unlikely by ultra deep sequencing of the *MLH1* variant in DNA isolated from normal mucosa, blood, urine and saliva. Although initially being suspect for Lynch syndrome it was eventually concluded that a metachronously diagnosed colon carcinoma that metastasized to both ovaries was most likely.

## Introduction

In this report we describe a female patient diagnosed with bilateral endometrioid carcinoma of the ovaries at the age of 44. One year later an adenocarcinoma of the colon was detected. The discovery of the colon carcinoma created doubt about the primary origin of the ovarian tumors. Besides, because the patient met the Amsterdam/Bethesda revised criteria, Lynch syndrome (LS) was suggested.

The ovaries can be affected by metastases from several primary tumor sites [1]. Most metastases originate from the gastrointestinal tract, with the colon as most frequent primary location. However, primary ovarian tumors are more common than ovarian metastases; 85 versus 15% [2]. Since subtypes of primary ovarian cancers (especially endometrioid and mucinous adenocarcinomas) can show overlapping histological and immunohistochemical features with gastrointestinal tumor metastases, it can be difficult to discriminate these [3, 4]. A combined analysis of clinical and molecular features can be helpful in correctly diagnosing these tumors. Reanalyis of this case revealed both macroscopic and microscopic evidence for a colonic origin of the ovarian tumors. This thought was supported by up-to-date extensive molecular analyses that showed a clonal relationship between both tumors. Lynch syndrome, including DNA mismatch repair gene mosaicism, was ruled out.

## Materials and methods

### Immunohistochemistry

Immunohistochemistry was performed as previously described [5]. The antibodies and dilutions that were used are as follows: MSH2 (1:25; DAKO Santa Clara, United States), MSH6 (1:400; GeneTex Irvine, United States), PMS2 (1:80; DAKO Santa Clara, United States) and MLH1 (1:40; DAKO Santa Clara, United States), CDX2 (1:80; DAKO Santa Clara, United States), keratin-7 (1:400; DAKO Santa Clara, United States), keratin-20 (1:200; DAKO Santa Clara, United States), ER (1:40; DAKO Santa Clara, United States), PR (1:400; DAKO Santa Clara, United States) and vimentin (1:1000; DAKO Santa Clara, United States).

### Methylation specific assay

The promoter region of *MLH1* was analyzed by methylation-specific multiplex ligation-dependent probe amplification (MS-MLPA) as previously described [6].

### Microsatellite instability (MSI) analysis

Microsatellite analysis was performed using five mononucleotide microsatellite markers as previously described [7].

### Germline analysis

Germline analysis of *MLH1, PMS2, MHS2* and *MSH6* variant was performed on DNA isolated from lymphocytes from a blood sample using standard procedures including the analysis for large deletions/duplications by the multiplex ligation-dependent probe amplification (MRC Holland, the Netherlands).

### Somatic and mosaicism analysis

Somatic mutation analysis of *MLH1* was performed using a laboratory developed multiplex AmpliSeq based NGS protocol followed by confirmation of detected mutations by Sanger sequencing.

Additional analysis of somatic variations was performed on DNA isolated using a fully automated DNA extraction procedure. The concentration of DNA was measured using a fluorometer (Qubit dsDNA HS, Thermo Fischer Scientific, Waltman MA USA). The amplicon library for targeted sequencing was constructed using AmpliSeq Cancer Hotspot Panel v2. This panel consists of a single primer pool and is designed to detect somatic cancer hotspot pathogenic variants in 207 amplicons covering 50 cancer related genes, including genes as *APC, KRAS, TP53, SMAD4* that are often altered in colorectal cancer. The whole *APC* gene was analyzed in a separate analysis as in the cancer hotspot panel only the mutation cluster region of *APC* is covered. Mosaicism analysis of the identified *MLH1* variant was performed by using a panel covering *MSH2, MSH6, PMS2, MLH1, POLD1* and *POLE*. Libraries were prepared with 10 ng of genomic DNA, and each sample was uniquely barcoded using IonXpress barcodes (Life Technologies). Next-generation sequencing was carried out according to the Ion Proton protocol.

### Bioinformatic analysis

The unaligned BAM file generated by the Proton sequencer were mapped against the human reference genome (GRCh37/hg19) using the TMAP 5.0.7 software with default parameters (https://github.com/iontorrent/TS). Subsequently variant calling was done using the Ion Torrent specific caller, Torrent Variant Caller (TVC)-5.0.2, using the recommended Variant Caller Parameter for Cancer Hotspot Panel v2. Variant interpretation was done using Geneticist Assistant (Softgenetics) which assigns Functional Prediction, Conservation scores and Disease associated information to each variant (http://softgenetics.com/GeneticistAssistant_2.php). Once pathogenicity is assigned to a variant, the same pathogenicity is automatically attributed the next time the variant is observed. Integrative Genomics Viewer (IGV) was used for visually inspecting variants (doi: 10.1093/bib/bbs017). The analysis of the complete *APC* gene was performed as described previously [8]. LOH was analyzed by comparison of variant and wild type DNA reads of the NGS results.

## Case report

**Fig. 1:**
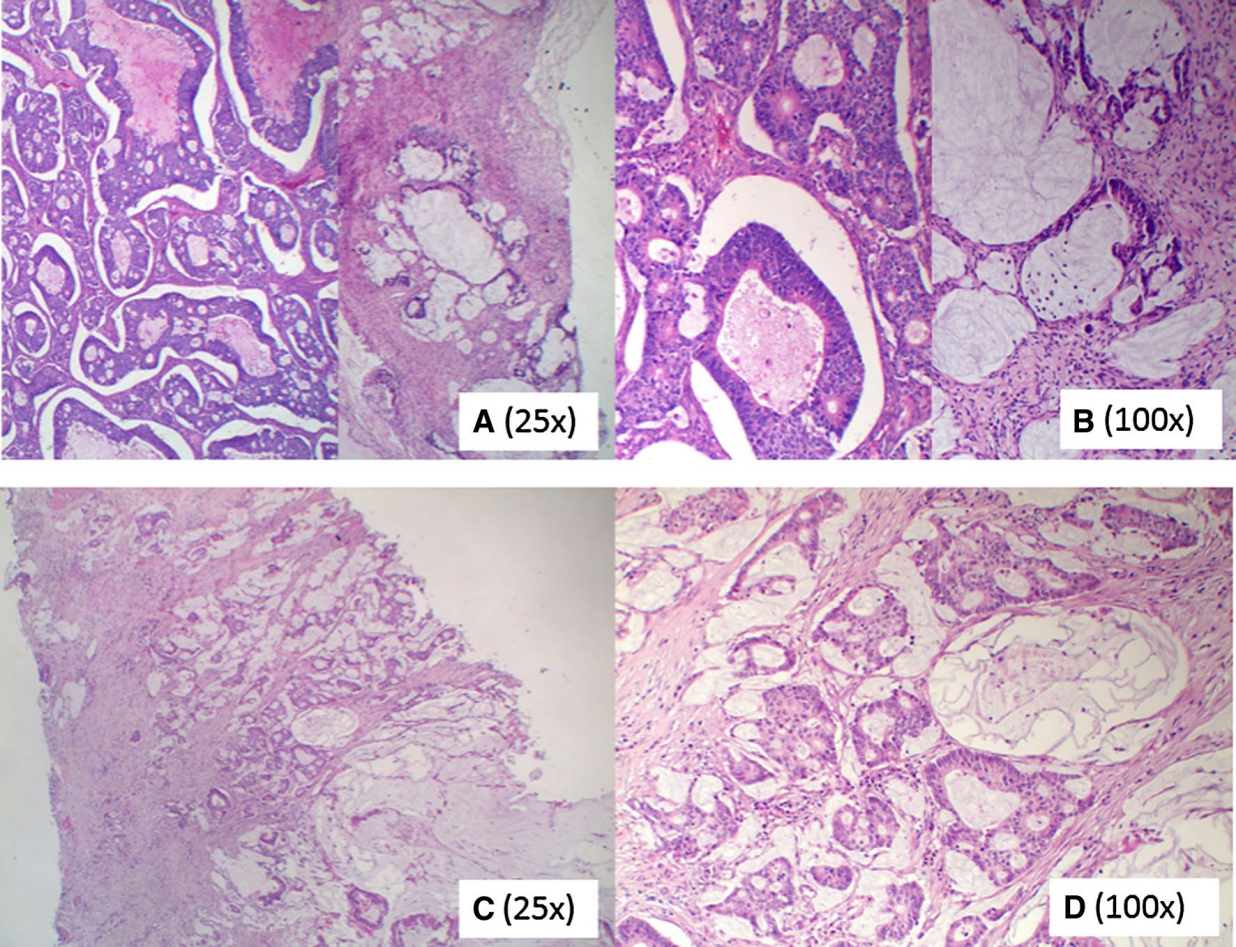
Shows the histological picture of the ovarian tumor (**a, b**) and the colon tumor (**c, d**). In **a**, **b** both endometrioid and mucinous parts of the ovarian tumor are shown

We describe a female patient with a family history of ovarian cancer (one sister at the age of 56 years), breast cancer (one sister at the age of 59 years), colon cancer (patient’s mother at the age of 80 years) and a (non melanoma) skin cancer (the sister diagnosed with breast cancer). The index patient had one hyperplastic polyp removed from the rectum at the age of 43. Aged 44, she was diagnosed with bilateral endometrioid carcinoma of the ovaries with focally mucinous differentiation (Fig. 1), clinical stage 1B according to the FIGO staging system. Surgery was performed and she was treated with adjuvant chemotherapy comprising a regimen of cyclofosfamide and carboplatin. At age 45, 12.5 months later, she was diagnosed with an adenocarcinoma of the colon, treated by a left-sided hemicolectomy. Based on these clinical records the patient met the Amsterdam/Bethesda revised criteria. Patient has remained disease-free until the age of 64. However, the discovery of the (mucinous) colon carcinoma showing partly a similar morphology as the ovarian tumors (Fig. 1), created doubt about the primary origin of the ovarian tumors. Lynch syndrome (LS), in which independent ovarian and colon tumors had developed, was suggested.

Immunohistochemistry testing of the MMR proteins MLH1, PMS2, MSH2 and MSH6 of the colonic and ovarian tumors showed DNA MMR deficiency with loss of expression of MLH1 and PMS2. Microsatellite instability (MSI) testing using mononucleotide microsatellite markers showed an MSI-H phenotype. A sporadic origin of these MMR deficient tumors due to *MLH1* promoter hypermethylation was excluded. Our patient was subsequently referred to a clinical geneticist. However LS could not be confirmed after negative lymphocyte DNA testing of *MLH1, PMS2, MHS2* and *MSH6* for germline pathogenic variants. Also germline testing of *BRCA1* and *BRCA2* in two sisters of the patient was negative.

Reevaluation of the metachronously diagnosed colon tumor confirmed the primary origin in the colon as the bulk of the tumor was bulging in the colonic lumen. Furthermore, the serosal lining was unaffected. Immunohistochemical stainings of the ovarian tumor showed a phenotype compatible with a metastasis from a colon tumor (keratin-7 negative / keratine-20 and CDX-2 positive). ER, PR and vimentin were also negative. However, ovarian tumors with mucinous differentiation can show a wide variety of keratine-7/keratin-20 immunoprofile patterns, and should be interpreted with caution [9].

**Fig. 2:**
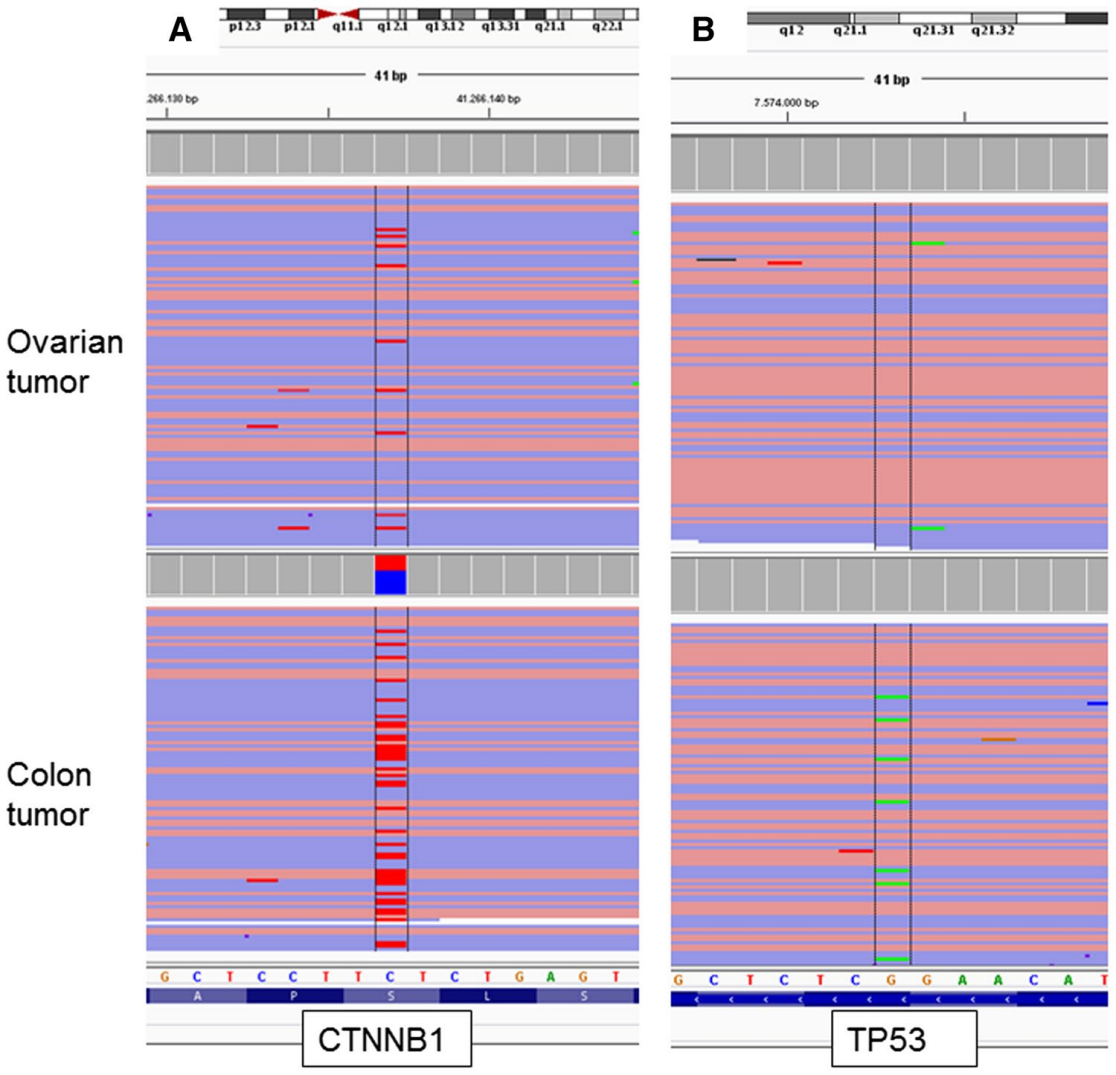
**a** Shows the reads including the pathogenic *CTNNB1* variant that is present in both the ovarian and the colon tumor. **b** Shows the reads including the pathogenic *TP53* variant that is present in the colon tumor, but not in the ovarian tumor

Somatic testing (Table 1) of *MLH1* showed an identical *MLH1* class 5 pathogenic variant (c.1624C > T, p.(Gln542*)) in both colon and ovarian tumors. Next, loss of heterozygosity for *MLH1* was shown by absence of the WT(wild type)-allele. We also somatically tested the complete *APC* gene for pathogenic variants in these lesions, as finding of pathogenic *APC* variants in ovarian neoplasms would point at a colonic origin of the lesions. No *APC* variants were found, however an identical activating class 5 *CTNNB1* pathogenic variant (c.134C > T; p.(Ser45Phe)) was identified, the molecular alternative for Wnt-pathway activation (Fig. 2a). Finding identical *MLH1* and *CTNNB1* variants would suggest a clonal relation between the colon and ovarian tumor. Additionally, a class 5 pathogenic *TP53* variant (c.1024C > T, p.(Arg342*)) was detected in the colon tumor, but absent in the ovarian tumor (Fig. 2b). In order to estimate putative germline mosaicism we performed ultra-deep sequencing of the *MLH1* (c.1624C > T, p.(Gln542*)) variant in DNA isolated from normal colonic mucosa, saliva, blood and urine. All isolates showed sufficient (> 10 K) coverage, but showing no presence of the *MLH1* variant, rendering germline mosaicism unlikely. It was concluded that a metachronously diagnosed colorectal tumor that metastasized to both ovaries was the most likely diagnosis.

**Table 1:** Pathogenic variants, promoter methylation status and immunohistochemical expression of mismatch repair genes in the ovarian tumor, colon tumor and leukocyte DNA

Gene	Ovary (T%: > 60%)	Colon (T%: > 50%)	Lymphocytes
*TP53*	No pathogenic variant	c.1024C > T, p.(Arg342*)/ 6,1% mutant reads	
*MLH1*	c.1624C > T, p.(Gln542*)/ 76% mutant reads LOH Loss of expression by IHC No promoter hypermethylation	c.1624C > T, p.(Gln542*)/ 52% mutant reads LOH Loss of expression by IHC No promoter hypermethylation	No pathogenic variant
*CTNNB1*	c.134C > T, p.(Ser45Phe)/ 9,2% mutant reads	c.134C > T, p.(Ser45Phe) 38% mutant reads	
*PMS2*	Loss of expression by IHC	Loss of expression by IHC	No pathogenic variant
*MSH2*	Normal expression by IHC	Normal expression by IHC	No pathogenic variant
*MSH6*	Normal expression by IHC	Normal expression by IHC	No pathogenic variant
*APC*	No pathogenic variant	No pathogenic variant	

The table shows an overview of the detected pathogenic variants, methylation assays and immunohistochemical staining results of mismatch repair genes in one of the ovarian tumors, the colon tumor and DNA isolated from blood

*LOH* loss of heterozygosity, *IHC* immunohistochemical staining, *T%* tumor cell percentage

## Discussion

In the current report we address a remarkable clinical dilemma once metachronous ovarian and colon tumors are diagnosed and the possibility of a Lynch syndrome needs to be answered. The female patient we now present with bilateral ovarian cancer was treated as having primary bilateral ovarian cancer. However, only 12.5 months after the first diagnosis the true primary origin of these lesions was questioned with the resection of a DNA mismatch repair deficient left sided colon cancer. After reevaluation and molecular analysis a clonal relation was identified between the ovarian and colonic lesions. As MMR deficient cancers mostly lack distant metastases possibly due to the interaction with the immune system, it is noteworthy that this DNA MMR deficient colon cancer probably metastasized to the ovaries [10].

About 15% of all ovarian tumors turn out to be metastases [2]. Histological parameters are not always sufficient to discriminate between a primary tumor and/or metastasis. Nowadays, molecular analysis can be a helpful tool to make this distinction in selected cases [11]. Inactivating *APC* pathogenic variants are almost exclusively found in colon tumors. Thus, the presence of a pathogenic variant is a strong argument for a primary colon tumor [3, 11, 12]. In our patient no pathogenic variants in the *APC* gene were found, but an identical activating *CTNNB1* variant was present in both ovary and colon tumors. As *CTNNB1* variants are very rare in colon carcinomas, this might suggest the ovarian tumor as the primary origin [13]. On the other hand activating *CTNNB1* pathogenic variants are often found in colon cancer associated with DNA mismatch repair deficiency [14–16]. Only an incidental report of metastatic mismatch repair deficient colon carcinoma to the ovaries is described [17]. In previous published research we did not find any *CTNNB1* pathogenic variants in MMR proficient colorectal metastases to the ovary [11]. With respect to the ovary, *CTNNB1* pathogenic variants have mainly been found in endometrioid ovarian cancers [13]. However, the histopathological findings in our case do not suggest metastases from the ovary since the colonic tumor was located at the luminal site. In case of a metastasis the bulk of the tumor would have been present on the serosal site. Besides, ovarian cancers metastasizing to the colon, and morphologically mimicking a primary colon tumor are probably very rare. Furthermore in case of bilateral ovarian tumors the odds favor metastases from a primary tumor elsewhere in the body.

In our patient the same somatic *MLH1* pathogenic variant and concomitant loss of heterozygosity of the wild type allele was present in the ovarian and colon cancer. As the detected *MLH1* variant was not found by deep sequencing of DNA isolated from normal mucosa, saliva, blood and urine a germline mosaicism was rendered unlikely.

Somatic *MLH1* pathogenic variants in sporadic tumors are mainly associated with gastrointestinal tumors [13, 18]. *MLH1* pathogenic variants are not commonly found in ovarian cancer, although one study found *MLH1* pathogenic variants in 8.7% epithelial ovarian cancer [19]. Usually, *TP53* pathogenic variants occur early in the evolutionary development of a tumor. Our patient’s tumors showed in two tumors *CTNNB1* as well as *MLH1* pathogenic variants, but only in the colon tumor a *TP53* pathogenic variant was identified. The presence of this variant can be explained by tumor progression within the primary colon tumor. Apparently in this case, the pathogenic *TP53* variant is not present in the metastasizing clone. Such spatial differences in mutation profiles within a tumor are known as intra-tumor heterogeneity.

In summary, we discuss the clinical dilemma with metachronous diagnosed bilateral mismatch repair deficient ovarian and colon cancer harboring a pathogenic MMR variant. In our case Lynch syndrome as well as a postzygotic somatic mutation leading to mosaicism of multiple normal tissues are very unlikely. Molecular analysis showed a clonal relationship between the ovarian and colon tumors with histopathological analysis suggesting the colon tumor being the primary tumor.
